# On the Metal Aluminum

**Published:** 1856-04

**Authors:** H. L. Burpee

**Affiliations:** Brooklyn


### Selected Articles.

### ON THE METAL ALUMINUM.

M. Wœhler having contested the priority of the extraction of the metal aluminum from alumina with M. Deville, the latter has replied in a paper before the French Academy urging that the metal he has obtained by sodium and by using new apparatus, differs essentially in the distinctness of its reaction from the aluminum of M. Wœhler. This difference is due to impurities which can not possibly be removed when the operation is made in platina vases: and he asserts that he has ascertained by minute analysis that the aluminum prepared according to M. Wœhler's method contains soda and the platina: now platina raises the point of fusion of the alloy, and the sodium takes from it its most precious properties; making it subject to the influence of boiling water and the weak acids, while pure aluminum resists them; further imperceptible but pure globules have remained three months in sulphuric acid or weak nitric acid, and have not yet been in the least degree changed, and in boiling nitric acid the dissolution proceeds so slowly that M. Deville was forced to abandon that method of analysis; lastly, if a globule of pure aluminum was dropped on red hot and melted caustic soda, it resisted that energetic agent. The aluminum employed in these experiments (it has been analyzed) was perfectly pure, and it is upon these properties, joined to its inalteration when exposed to the air, that M. Deville grounds his hopes to make it useful. It is also worth attention that while M. Wœhler obtained merely microscopical globules, M. Deville now produces masses of it whose volume is limited only by the quantity of matter employed. He ended this reply by suggesting that other more common metals than aluminum, are perhaps less known than may be thought, and he expressed the hope that when he shall have completed a memoir on the pure metals produced and melted by certain yet secret processes, which he has long been preparing, he shall exhibit some unexpected results. Thus he instanced cobalt and nickel, which possess useful physical properties, such as malleability, ductility, developed to a most extraordinary degree; further they enjoy a tenacity far exceeding that of iron, which hitherto has passed as the most tenacious metal: for, according to the experiments made by M. Werthiem on these metals, the weights which determine the rupture of wires of iron, cobalt and nickel of the same dimensions, are 60 for iron, 115 for cobalt, and 90 for nickel, which shows the tenacity of cobalt double that of iron; besides, nickel and cobalt are worked at the forge with the same facility as iron, are oxydized less easily than iron, and are susceptible of being employed in the same manner as iron.

The following is an abstract of a report presented to the French Academy on the subject of aluminum and its preparation:

"The thorough working of aluminum by means of the chloride of this metal and sodium is, by general admission, a great acquisition to science. M. Deville procured chloride of aluminum by causing the chlorine to react on a mixture of alumina and coal-tar, previously calcined. The operation was effected in a gas retort with extraordinary facility and perfection. The result of M. Deville's observations is, that the action of the chlorine is procured upon a layer of one, or at most two, decimeters of the mixture, so that the absorption of the gas is always complete. The condensation of chloride of aluminum is effected in a chamber of masonry lined with tiles. This chloride is so compact that it can be seen on the table, of considerable density, and composed of yellow crystals. Very slightly ferruginous, it is purified completely during the process of extracting the aluminum, in which its vapor passes over iron filings heated to 400 deg. C. or thereabout. The sesqui-chloride of iron, as volatile as the chloride of aluminum, is changed by contact with the iron, and becomes comparatively very fixed. The vapor of the chloride of aluminum rising from the apparatus forms colorless and very transparent crystals. Sodium is being prepared the meanwhile in large and small vessels with remarkable facility. Having studied with particular care the influence of temperature on the surfaces exposed to heat, and the activity of the vapor of sodium as it escapes from the apparatus, M. Deville is convinced that, by properly regulating the relation between the heated surfaces and the section of tubes from which the sodium issues, one could procure this metal at a moderately high temperature, about that, perhaps, of the melting point of silver. Already even the cylinders are much less heated than are the vessels used in the manufacture of zinc. The author is now employed in producing sodium in continuous apparatus.

"As to the reaction of the chloride of aluminum upon the sodium, that is done in metallic tubes, the form and management of which are not yet sufficiently scientific; in this particular there is yet something deficient. It will be remarked, undoubtedly, that in the details above there is no mention of the very reduced price which Messrs. Dumas and Balard have promised. It appears that, for the present, at least, this price is still very high, and very far from being what would be considered the net cost, as stated by us conditionally, of the agents necessary to extract the aluminum. Moreover, M. Dumas has not explained himself formally concerning the price even of the new metal, and he has anticipated too much in this respect. Now, if sodium, which cost lately 1,000 francs a kilogram, should rise 30 francs more, as it requires three times the quantity of that to extract a portion of aluminum, it will be perceived that this latter article would not be so accessible as they would have it seem, not to mention other particulars which increase the expense. Still there is reason to believe that a factory of aluminum established at Marseilles, turning to account the chlorine of muriatic acid, which is produced in superabundance, and the aluminum of certain deposits in the vicinity, could offer this precious metal at a price sufficiently low to place it shortly among the more common ones.

"Scarcely has aluminum been ranked among metals before, independently of the unexpected service it will render as such to the arts and sciences, it begins to figure as an electro-chemical agent in a no less remarkable manner. The director of the galvano-plastic department of the Mint, M. Hulot, submitted to the Academy, through M. Dumas, some assays, in which this metal was substituted for platina as an electro-negative element in the piles to a single liquid. M. Hulot has succeeded, not without great difficulty, in rolling perfectly, and without loss, an ingot of aluminum of thirty and some gram's, procured from one of the first meltings of this metal obtained by M. Rousseau—less white than at first, and containing traces of iron and silicium. A connection of aluminum and zinc, amalgamated for a long time, charged with water acidulated with a twentieth of sulphuric acid at 66 deg., disengaged during the first hours considerable hydrogen, and produced a current equal, if not superior, to that of a connection of platina and zinc excited to the same degree. The author asserts that the next day the electro-motive force of the pile was reduced nearly one-quarter; but he remarks that it suffices to immerge the aluminum in nitric acid, or still better, as it appears, in sulphuric acid—as it is expedient to effect it at once—to give to the circle its first force.

"As aluminum is nine times lighter than platina, and presents also a surface nine times more extended than the latter metal, with an equal thicknés, its substitution for platina should be productive of real advantages, above all now that its price has become very accessible. The aluminum here spoken of is very difficult to forge. In order to roll it, it has been found necessary to anneal it at each pass. By decomposing copper electro-chemically on a plate of aluminum, they have succeeded, by the aid of rollers, in reducing it to very thin plates. Hard aluminum acquires by annealing an inflexibility which would make it of great use in the suspension of all kinds of scales for assays or analysis. This metal is so light that, the weights of the system being the same, the arms of the beam can be elongated a great deal, and long blades can be placed even on the extreme points of suspension, as on the centre of oscillation. The author does not doubt that, in weighing 20 grammes, the sensibility of the balance would not rise a half-millionth."

The price of aluminum, a short time since, in France, was about the rate of gold. M. Dumas, in a recent communication to the academy, stated that, owing to recent discoveries reducing the expense of extracting it, the cost of production was now about one hundred times less; and M. Balard, another member, stated that there was little doubt that the effect of competition in its manufacture, together with the advantages of throwing it open to the industrial resources of the world, would be to reduce the price as low as five francs the kilogram, or about forty cents a pound.

This important result is mainly attributable to the facility with which we are now able to procure pure sodium in abundance, which is the active agent for the revivication of aluminum, and which was at one time very expensive. M. Dumas observes that the generalization of the procedure of M. Deville, the application of chlorine to the extraction of metals, forms a new era in metallurgy. Among the many remarkable qualities of aluimnum, such as its resistance to oxydation, either in the air or by acids, its hardness, its wonderful lightness, its malleableness, the facility of molding it, &c., M. Dumas mentions another—its sonority. An ingot was suspended by a string, and, being lightly struck, emitted the finest tones, such as are obtained only by a combination of the best metals.—*Dental Recorder.*

